# Genetic heterogeneity of patients with suspected Silver-Russell syndrome: genome-wide copy number analysis in 82 patients without imprinting defects

**DOI:** 10.1186/s13148-017-0350-6

**Published:** 2017-05-15

**Authors:** Takanobu Inoue, Akie Nakamura, Tomoko Fuke, Kazuki Yamazawa, Shinichiro Sano, Keiko Matsubara, Seiji Mizuno, Yoshika Matsukura, Chie Harashima, Tatsuji Hasegawa, Hisakazu Nakajima, Kumi Tsumura, Zenro Kizaki, Akira Oka, Tsutomu Ogata, Maki Fukami, Masayo Kagami

**Affiliations:** 1 Department of Molecular Endocrinology, National Research Institute for Child Health and Development, 2-10-1, Okura Setagaya-ku, Tokyo, 157-8535 Japan; 2 Department of Pediatrics, The University of Tokyo Hospital, 7-3-1 Hongo, Bunkyo-ku, Tokyo, 113-8655 Japan; 3 Department of Pediatrics, Central Hospital, Aichi Human Service Center, 713-8 Kagiya-cho, Kasugai, Aichi 480-0392 Japan; 4 Department of Pediatrics, The Japan Baptist Hospital, 47 Yamanomoto-cho, Kitashirakawa, Sakyo-ku, Kyoto, 606-8273 Japan; 5 Department of Pediatrics, Kyoto Prefectural University of Medicine, 465 Kajii-cho, Kamigyo-ku, Kyoto, 602-8566 Japan; 6 Tsumura Family Clinic, Kumi Shounika, 858-1 Watarihashi-cho, Izumo, Shimane 693-0004 Japan; 7 Department of Pediatrics, Japanese Red Cross Kyoto Daiichi Hospital, 15-749 Honmachi Higashiyama-ku, Kyoto, 605-0981 Japan; 8 Department of Pediatrics, Hamamatsu University School of Medicine, 1-20-1 Handayama, Higashi-ku, Hamamatsu, Shizuoka 431-3192 Japan

**Keywords:** Silver-Russell syndrome, Pathogenic copy number variation, Array comparative genomic hybridization, Netchine-Harbison clinical scoring system, 4p microdeletion syndrome, Mosaic trisomy 18, 19q13.11 deletion syndrome, Williams syndrome

## Abstract

**Background:**

Silver-Russell syndrome (SRS) is a rare congenital disorder characterized by pre- and postnatal growth failure and dysmorphic features. Recently, pathogenic copy number variations (PCNVs) and imprinting defects other than hypomethylation of the *H19*-differentially methylated region (DMR) and maternal uniparental disomy chromosome 7 have been reported in patients with the SRS phenotype. This study aimed to clarify the frequency and clinical features of patients with SRS phenotype caused by PCNVs.

**Methods:**

We performed array comparative genomic hybridization analysis using a catalog array for 54 patients satisfying the Netchine-Harbison clinical scoring system (NH-CSS) (SRS-compatible) and for 28 patients presenting with three NH-CSS items together with triangular face and/or fifth finger clinodactyly and/or brachydactyly (SRS-like) without abnormal methylation levels of 9 DMRs related to known imprinting disorders. We then investigated the clinical features of patients with PCNVs.

**Results:**

Three of the 54 SRS-compatible patients (5.6%) and 2 of the 28 SRS-like patients (7.1%) had PCNVs. We detected 3.5 Mb deletion in 4p16.3, mosaic trisomy 18, and 3.77–4.00 Mb deletion in 19q13.11-12 in SRS-compatible patients, and 1.41–1.97 Mb deletion in 7q11.23 in both SRS-like patients. Congenital heart diseases (CHDs) were identified in two patients and moderate to severe global developmental delay was observed in four patients.

**Conclusions:**

Of the patients in our study, 5.6% of SRS-compatible and 7.1% of SRS-like patients had PCNVs. All PCNVs have been previously reported for genetic causes of contiguous deletion syndromes or mosaic trisomy 18. Our study suggests patients with PCNVs, who have a phenotype resembling SRS, show a high tendency towards CHDs and/or apparent developmental delay.

## Findings

### Background

Silver-Russell syndrome (SRS) is a rare congenital disorder characterized by pre- and postnatal growth failure and dysmorphic features [1]. The diagnosis of SRS is based on a combination of clinical features. Several clinical scoring systems for SRS have been proposed [2–8]. Of these, the Netchine-Harbison clinical scoring system (NH-CSS) is the most sensitive and has the highest negative predictive value [8]. NH-CSS includes six items: (1) small for gestational age (birth weight and/or birth length ≤ −2 standard deviation score (SDS) for gestational age); (2) postnatal growth retardation (height at 24  ± 1 months ≤ −2 SDS or height ≤ −2 SDS below mid-parental target height); (3) relative macrocephaly at birth (head circumference at birth ≥1.5 SDS above birth weight and/or length SDS); (4) protruding forehead (forehead projecting beyond the facial plane from the side view among toddlers); (5) body asymmetry (leg length discrepancy (LLD) of ≥0.5 cm or arm asymmetry or LLD < 0.5 cm with at least two other asymmetrical body parts (one non-face)); (6) feeding difficulties and/or low body mass index (BMI) (BMI ≤ −2 SDS at 24 months or current use of a feeding tube or cyproheptadine for appetite stimulation). When patients meet four or more of the six NH-CSS items, they are clinically diagnosed with SRS [8]. Furthermore, additional clinical features such as triangular face, fifth finger clinodactyly, and brachydactyly were frequently identified in SRS patients [9], but these clinical features are not included in the NH-CSS items.

The most common genetic causes of SRS are hypomethylation of the *H19*-differentially methylated region (DMR) at the 11p15 imprinted region (*H19*-hypo) and maternal uniparental disomy chromosome 7 (UPD(7)mat) [1]. Recently, Azzi et al. reported that *H19-*hypo and UPD(7)mat were detected in 58.3 and 18.3% of patients, respectively, satisfying the NH-CSS criteria [8]. In addition, 11p15 duplication and other imprinting disorders such as Temple syndrome, maternal uniparental disomy for chromosome 16 and 20, and pathogenic copy number variations (PCNVs) are less frequently identified in patients with SRS clinical phenotypes [8, 10].

Several reports have described patients with the SRS phenotype caused by PCNVs, but these reports have the following weak points: (1) insufficient clinical information on the patients; (2) imprinting disorders other than SRS were not excluded, and (3) the number of patients involved in these studies was small [6, 8, 11–13]. To clarify the frequency and the clinical features of patients with the SRS phenotype caused by PCNVs, we performed molecular and clinical studies in patients with suspected SRS who had normal methylation levels for 9 DMRs related to known imprinting disorders.

## Methods

### Patients

**Fig. 1:**
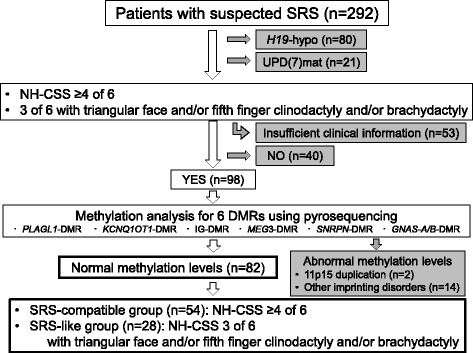
The flowchart of inclusion criteria. From 2002 to 2016, 292 patients were referred to us for genetic diagnosis of Silver-Russell syndrome (SRS). We studied 82 patients with SRS clinical features, and no abnormal methylation levels for 9 differentially methylated regions related to known imprinting disorders.

We summarized the inclusion criteria in Fig. 1. We studied 82 out of 292 patients referred to us for genetic diagnosis of SRS from 2002 to 2016. All 82 patients had normal methylation levels for 9 DMRs related to known imprinting disorders, that is, *H19*-DMR, *PEG1/MEST*-DMR, *PEG10*-DMR, *PLAGL1-*DMR, *KCNQ1OT1*-DMR, IG-DMR, *MEG3*-DMR, *SNRPN-*DMR, and *GNAS*-*A/B*-DMR. Methylation analysis was performed by combined bisulfite restriction analysis or pyrosequencing as previously reported [9, 14]. Fifty-five out of 82 patients were examined using karyotyping before being referred to us and were confirmed to have normal karyotypes. All patients were Japanese except for two patients who were from Canada and the USA.

We collected clinical information from the attending physicians by questionnaire. Fifty-four patients who satisfied ≥ 4 out of 6 NH-CSS criteria were included in this study and were classified as SRS-compatible. Furthermore, because some patients who did not satisfy NH-CSS criteria had *H19*-hypo or UPD(7)mat [8, 15–17], 28 patients who presented three NH-CSS items together with triangular face and/or fifth finger clinodactyly and/or brachydactyly, which are common clinical features in SRS patients [9], were also included in this study and were classified as SRS-like. For patients under 23 months old, the score for postnatal growth retardation was excluded from the NH-CSS criteria. When these patients satisfied four or five NH-CSS items or three NH-CSS items together with triangular face and/or fifth finger clinodactyly and/or brachydactyly, we classified them as SRS-compatible or SRS-like, respectively.

### Molecular studies

We extracted leucocyte genomic DNA from peripheral blood using the Gentra Puregene Blood Kit (Qiagen, Hilden, Germany). We then performed array comparative genomic hybridization analysis using a 60 K catalog array (Agilent Technologies, Palo Alto, CA, USA) in 82 patients with normal methylation levels in 9 DMRs. To detect PCNVs, we first extracted CNVs that had three or more continuous probes with aberrant signals. Next, we evaluated whether these CNVs were benign or pathogenic. In our study, the CNVs reported in the Database of Genomic Variants [18] as a normal variant were considered to be nonpathogenic, while CNVs that have been previously reported to cause some of the known syndromes were considered to be pathogenic. In cases where CNVs were not reported in the database or medical publications, we searched whether the genes involved in the regions were associated with the SRS phenotype. Karyotyping was performed when necessary.

### Clinical studies

We compared the clinical features between our patients with PCNVs and patients with genetic syndromes caused by the same PCNVs. Furthermore, we calculated the frequencies of congenital heart diseases (CHDs), motor developmental delay, and speech delay in patients with PCNVs. Echocardiography and tests for intelligence quotient or developmental quotient were performed in all patients with PCNVs.

### Statistical analysis

The frequencies of clinical feature differences between patients with PCNVs, *H19*-hypo, and UPD(7)mat were analyzed by Fisher’s exact probability test using a statistical software program (StatFlex for Windows Ver.6.0, Artec, Osaka, Japan). *P* < 0.05 was considered significant.

## Results

### Molecular studies

**Fig. 2:**
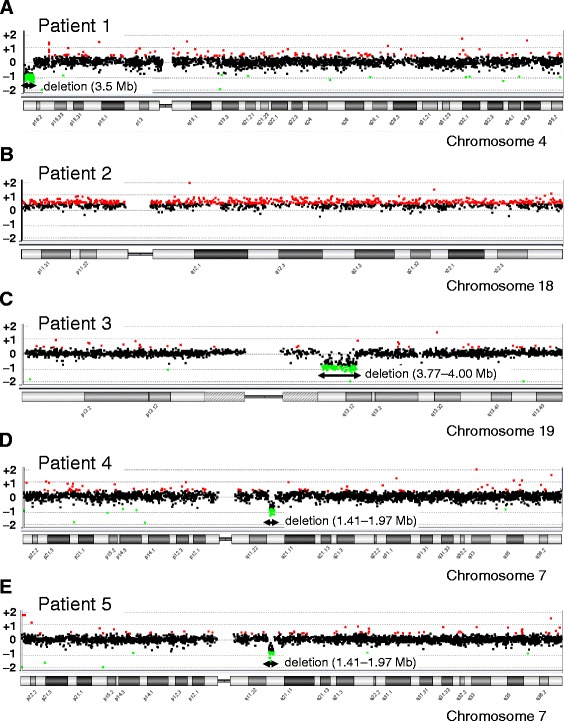
Array comparative genomic hybridization profiles of the five patients with pathogenic copy number variations. **a** Patient 1 (SRS-compatible, 4p16.3 deletion). **b** Patient 2 (SRS-compatible, mosaic trisomy 18). **c** Patient 3 (SRS-compatible, 19q13.11-12 deletion). **d** Patient 4 (SRS-like, 7q11.23 deletion). **e** Patient 5 (SRS-like, 7q11.23 deletion). The *black*, *red*, and *green dots* denote signals indicative of the normal, increased (> + 0.4), and decreased (<–0.8) copy numbers, respectively

We identified 3 patients (patients 1–3) with PCNVs from 54 patients in the SRS-compatible group (5.6%), and 2 patients (patients 4 and 5) with PCNVs from 28 patients in the SRS-like group (7.1%) (Table 1). We detected 3.5 Mb deletion in 4p16.3 including the critical region of 4p microdeletion syndrome in patient 1 [19] (Fig. 2a), trisomy 18 in patient 2 (Fig. 2b) and 3.77–4.00 Mb deletion in 19q13.11-12 involving the smallest region of overlap of 19q13.11 deletion syndrome [20] in patient 3 (Fig. 2c). Subsequently, we carried out karyotyping using peripheral blood and ascertained mosaic trisomy 18 (46,XY [14]/47,XY,+18 [6]) in patient 2. In the SRS-like group, we detected the same copy number change (1.41–1.97 Mb deletion in 7q11.23), which included the critical region of Williams syndrome [21] in patients 4 and 5 (Fig. 2d for patient 4, 2e for patient 5). Both parents of patients 1, 3, 4, and 5 did not present with the PCNVs detected in their children.

**Table 1:** Clinical features of three SRS-compatible and two SRS-like patients with PCNVs

	SRS-compatible			SRS-like	
Patient	Patient 1	Patient 2	Patient 3	Patient 4	Patient 5
Genetic cause	4p16.3 deletion	Mosaic trisomy 18	19q13.11-12 deletion	7q11.23 deletion	
Karyotype	46,XX	46,XY [14]/47,XY,+18 [6]	46,XX	46,XY	NE
Sex	Female	Male	Female	Male	Female
Present age (years)	9	1 9/12	4	19	7
Gestational age (weeks:days)	34:1	40:4	34:3	40:0	40:5
Birth length in cm (SDS)^a^	38 (−2.37)	41 (−4.60)	37.5 (−2.62)	45 (−2.36)	45 (−2.61)
Birth weight in g (SDS)^a^	1246 (−3.13)	1700 (−4.80)	1156 (−3.67)	2734 (−1.45)	2276 (−2.91)
Birth OFC in cm (SDS)^a^	27.0 (–2.17)	31.0 (−1.96)	24.0 (−3.79)	32.5 (−0.69)	32.4 (−0.92)
Height at 24 months in cm (SDS)^b, c^	70 (−4.87)	・・・	74.8 (−3.63)	85.2 (−3.06)	72.3 (−3.66)
BMI at 24 months (SDS)^b, c^	−3.34	・・・	−4.18	+0.27	+1.00
Present height in cm (SDS)^b^	104.5 (−3.82) at 8 years	73.3 (−3.38)	86.1 (−3.28) at 3 10/12 years	156.4 (−2.38) at 16 years	106.0 (−2.64)
Present weight in kg (SDS)^b^	11.6 (−7.86) at 8 years	8.77 (−2.28)	8.9 (−5.06) at 3 years	50.8 (−1.25) at 16 years	20.3 (−0.58)
Present OFC in cm (SDS)^b^	46.1 (−4.29) at 8 years	47.8 (+0.20) at 1 7/12 years	42.5 (−3.96) at 2 years	Unknown	Unknown
GH treatment	−	−	3 1/12 years~	5 ~ 16 years	−
SGA^d^	+	+	+	+	+
Postnatal growth failure^c, e^	+	・・・	+	+	+
Relative macrocephaly at birth^f^	−	+	−	+	+
Protruding forehead	+	+	+	−	−
Body asymmetry	−	+	−	−	−
Feeding difficulties and/or low BMI	+	+	+	−	−
NH-CSS	4/6	5/5	4/6	3/6	3/6
Triangular face	+	+	+	+	+
Fifth finger clinodactyly	−	−	−	−	+
Fifth finger brachydactyly	−	−	+	+	+
Present characteristic features of genetic syndrome caused by PCNV	Pre- and postnatal growth failure, Protruding forehead, Tube feeding, Greek warrior helmet appearance, Severe global developmental delay, Atrial septal defect, Epilepsy, Hearing loss	Prenatal growth failure, Tube feeding, Ventricular septal defect	Pre- and postnatal growth failure, Slender habitus, Long face, Cutis aplasia (posterior occiput), Moderate global developmental delay	Pre- and postnatal growth failure, Long philtrum, Medial eyebrow flare, Moderate global developmental delay	Pre- and postnatal growth failure, Prominent lips with open mouth, Hearing loss, Fifth finger clinodactyly, Moderate global developmental delay
Absent characteristic features of genetic syndrome caused by PCNV	Distinct mouth, Short philtrum	Overlapping fingers, Rocker bottom feet, Apnea, Single umbilical artery, Prominent occiput, Microcephaly, Inguinal hernia Umbilical hernia, Cleft lip, Cleft palate	Microcephaly	Prominent lips with open mouth, Hearing loss, Fifth finger clinodactyly, Cardiovascular anomalies, Hypercalcemia, Periorbital fullness, Joint hypermobility, Soft lax skin	Long philtrum, Medial eyebrow flare, Cardiovascular anomalies, Hypercalcemia, Periorbital fullness, Joint hypermobility, Soft lax skin
Congenital heart disease	Atrial septal defect	Ventricular septal defect	−	−	−
Development					
Motor developmental delay	+	−	+	+	+
Age at head control (months)	Unknown	4	7	Unknown	3
Age at sitting without support (months)	−	6	9	Unknown	10
Age at walking without support (months)	−	14	26	Unknown	24
Speech delay	+	+	+	+	+
IQ/DQ (age at examination)	10 (9 years)	78 (1 9/12 years)	50 (2 10/12 years)	50 (8 years)	50 (5 years)
Other findings	Severe neonatal asphyxia, Periventricular leukomalacia	−	−	−	−

*SRS* Silver-Russell syndrome, *PCNV* pathogenic copy number variation, *NE* not examined, *SDS* standard deviation score, *OFC* occipitofrontal circumference, *BMI* body mass index, *GH* growth hormone, *SGA* small for gestational age, *NH-CSS* Netchine-Harbison clinical scoring system, *IQ* intelligence quotient, *DQ* developmental quotient

^a^Birth length, weight and OFC were evaluated by the sex- and the gestational age-matched Japanese reference data (http://jspe.umin.jp/medical/keisan.html).

^b^Postnatal height, BMI, weight and OFC were evaluated by the sex- and the age-matched Japanese reference data (http://jspe.umin.jp/medical/keisan.html) (http://jspe.umin.jp/medical/taikaku.html).

^c^If we did not get information at 24 ± 1 months, we used the data at the nearest measure available older than 25 months.

^d^Birth length and/or birth weight ≤–2 SDS.

^e^Height at 24 ± 1 months ≤–2 SDS or height ≤ –2 SDS below mid-parental target height. Mid-parental target height was calculated as follows: [(father’s height + mother’s height)/2] +6.5 cm for boys and –6.5 cm for girls.

^f^Head circumference at birth ≥1.5 SDS above birth length and/or weight SDS.

### Clinical studies

**Fig. 3:**
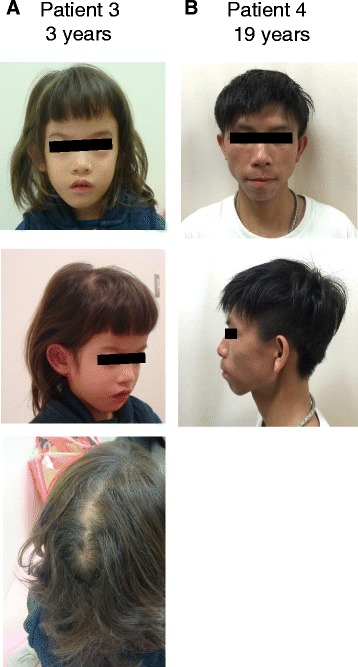
Photographs of patients with pathogenic copy number variations. **a** Patient 3 (SRS-compatible, 19q13.11 deletion syndrome). The patient had cutis aplasia over the occiput, which is characteristic of 19q13.11 deletion syndrome. **b** Patient 4 (SRS-like, Williams syndrome)

We summarized clinical features of patients with PCNVs, which are included in NH-CSS items and characteristic of each genetic syndrome caused by each PCNV, in Table 1. Patient 1 with 3.5 Mb deletion in 4p16.3 was born at 34 weeks of gestation with severe neonatal asphyxia and periventricular leukomalacia. This girl had four of the NH-CSS items, which are also identified in 4p microdeletion syndrome [19]. She had the characteristic features of 4p microdeletion syndrome such as Greek warrior helmet appearance, severe global developmental delay, an atrial septal defect, epilepsy, and hearing loss [19]. Patient 2 with mosaic trisomy 18 was 1-year-and-9-month-old boy, so we could therefore not evaluate his postnatal growth retardation using the NH-CSS. He presented with five other NH-CSS items and a ventricular septal defect, however, he did not show overlapping fingers and rocker bottom feet, which are characteristic features of trisomy 18 [22]. Patient 3 with 19q13.11-12 deletion had four NH-CSS items and some of the characteristic features of 19q13.11 deletion syndrome such as cutis aplasia over the posterior occiput [20] (Fig. 3a). This patient presented with moderate global developmental delay. Patients 4 and 5 exhibited triangular face and fifth finger brachydactyly and/or clinodactyly together with three NH-CSS items and moderate global developmental delay. However, they did not present many of the characteristic findings of Williams syndrome such as cardiovascular anomalies, hypercalcemia, and distinctive facial features [23] (Fig. 3b for patient 4). The attending physicians of all patients with PCNVs other than patient 1 were not clinical geneticists.

The frequencies of the clinical features in patients with PCNVs are shown in Table 2. CHDs were identified in two patients. Motor developmental delay and speech delay were observed in almost all patients with PCNVs.

**Table 2:** Phenotypical comparison between patients with PCNVs and *H19*-hypo or UPD(7)mat

	Patients with PCNVs in this study			Previous reports				*P* value	
	SRS-compatible	SRS-like	Total	*H19*-hypo		UPD(7)mat		PCNV vs. *H19*-hypo	PCNV vs. UPD(7)mat
SGA^a^	3/3 (100%)	2/2 (100%)	5/5 (100%)	43/43 (100%)	・・・	9/9 (100%)	・・・	1.0000	1.0000
Postnatal growth failure^b, c^	2/2 (100%)	2/2 (100%)	4/4 (100%)	29/35 (83%)	・・・	8/9 (89%)	・・・	0.6020	1.0000
Relative macrocephaly at birth^d^	1/3 (33%)	2/2 (100%)	3/5 (60%)	29/29 (100%)	・・・	7/9 (78%)	・・・	*0.0178*	0.5804
Protruding forehead	3/3 (100%)	0/2 (0%)	3/5 (60%)	31/37 (84%)	・・・	7/9 (78%)	・・・	0.2368	0.5804
Body asymmetry	1/3 (33%)	0/2 (0%)	1/5 (20%)	30/37 (81%)	・・・	3/9 (33%)	・・・	*0.0126*	1.0000
Feeding difficulties and/or low BMI	3/3 (100%)	0/2 (0%)	3/5 (60%)	16/34 (47%)	・・・	6/9 (67%)	・・・	0.6614	1.0000
Congenital heart disease	2/3 (67%)	0/2 (0%)	2/5 (40%)	・・・	8/145 (6%)^e^	・・・	0/17 (0%)^e^	*0.0361*	*0.0433*
Motor developmental delay	2/3 (67%)	2/2 (100%)	4/5 (80%)	18/37 (49%)^e^	・・・	6/9 (67%)^e^	・・・	0.3465	1.0000
Speech delay	3/3 (100%)	2/2 (100%)	5/5 (100%)	8/31 (26%)^e^	・・・	6/9 (67%)^e^	・・・	*0.0034*	0.2582
Reference				Fuke T, et al. [9]	Ghanim M, et al. [29]	Fuke T, et al. [9]	Ghanim M, et al. [29]		

*PCNV* pathogenic copy number variation, *H19-*hypo hypomethylation of the *H19*-differentially methylated region at the 11p15 imprinted region, *UPD(7)mat* maternal uniparental disomy chromosome 7, *SRS* Silver-Russell syndrome, *SGA* small for gestational age, *BMI* body mass index

^a^Birth length and/or birth weight ≤–2 standard deviation score (SDS) of the sex- and the gestational age-matched Japanese reference data (http://jspe.umin.jp/medical/keisan.html)

^b^Height at 24 ± 1 months ≤ –2 SDS of the sex- and the age-matched Japanese reference data (http://jspe.umin.jp/medical/keisan.html)

^c^If we did not get information at 24 ± 1 months, we used the data at the nearest measure available older than 25 months

^d^Head circumference at birth ≥1.5 SDS above birth length and/or weight SDS

^e^It is not clear that echocardiography and tests for intelligence quotient or developmental quotient were performed in all patients in these previous reports

Significant *P* values (<0.05) are italicized

## Discussion

We performed the largest genome-wide copy number analysis in 54 SRS-compatible patients and 28 SRS-like patients who had normal methylation levels for 9 DMRs related to imprinting disorders. PCNVs were identified in 5.6% of SRS-compatible patients and 7.1% of SRS-like patients. There are two studies about genome-wide copy number analysis in patients diagnosed as SRS using the NH-CSS. Azzi et al. identified 1 patient (9%) with PCNV (1q21 microdeletion) in 11 patients with 4 or more NH-CSS items who had neither *H19*-hypo, UPD(7)mat, *CDKN1C* mutation, imprinting abnormalities in other imprinted regions nor uniparental isodisomy other than UPD(7)mat [8]. Sachwitz et al. reported 2 patients (12%) with PCNVs (5q35 duplication and 4p16.3 deletion together with 7q36 duplication) in 17 patients satisfying NH-CSS criteria without *H19*-hypo, UPD(7)mat, gene mutations of *CDKN1C* and *IGF2*, and abnormal methylation levels in the 6q24, 14q32, and 20q13 imprinted regions [13]. Although there were differences in methods of molecular analysis and the number of the patients between our study and previous studies, the frequency of PCNVs in patients presenting the SRS phenotype without *H19*-hypo, UPD(7)mat and other imprinting disorders was similar among these studies.

PCNVs detected in this study have been previously reported to be responsible for 4p microdeletion syndrome, trisomy 18, 19q13.11 deletion syndrome, and Williams syndrome. To our knowledge, patients with suspected SRS have not been reported among patients with 4p microdeletion syndrome, 19q13.11 deletion syndrome, and Williams syndrome. A single case of mosaic trisomy 18 with suspected SRS had been previously reported [24]. This patient presented pre- and postnatal growth failure, poor sucking ability, slow weight gain, a relatively large head, body asymmetry of the chest and extremities, together with triangular face and fifth finger brachydactyly. Some NH-CSS items such as small for gestational age and feeding difficulties are also observed in patients with trisomy 18 [22]. Additionally, the clinical phenotype of mosaic trisomy 18 varies from the typical trisomy 18 phenotype to near-normal [25]. Actually, patient 2’s phenotype was not typical for trisomy 18, but this patient had some common overlapping features between SRS and trisomy 18. Thus, mosaic trisomy 18 patients may present with SRS clinical features. Some clinical features of 4p microdeletion syndrome overlap with SRS phenotype: both SRS and 4p microdeletion syndrome have pre- and postnatal growth failure, some facial features such as a protruding forehead [19]. Severe developmental delay, epilepsy, and hearing loss are characteristic of 4p microdeletion syndrome [19]. Because patient 1 presented with the latter three symptoms and also experienced severe neonatal asphyxia and neurological sequelae, the attending physician who was a clinical geneticist thought that the patient had SRS and neurological problems caused by asphyxia. These findings show the difficulties in clinically diagnosing SRS in patients with severe neurological complications. To our knowledge, the 19q13.11 deletion syndrome identified in patient 3 has been reported in only 25 patients and presents with pre- and postnatal growth retardation, slender habitus, severe postnatal feeding difficulties, microcephaly, motor- and intellectual developmental delay, hypospadias, and cutis aplasia over the posterior occiput [20, 26–28]. Because phenotypic overlaps exist between 19q13.11 deletion syndrome and SRS, we need to consider 19q13.11 deletion syndrome as a differential diagnosis of SRS. Patients 4 and 5 presented atypical clinical features of Williams syndrome. In addition, pre- and postnatal growth failure, fifth finger clinodactyly, which were detected in patients 4 and/or 5, are common clinical features between Williams syndrome and SRS [23]. Thus, Williams syndrome patients with atypical clinical features may be misdiagnosed with SRS.

We compared the frequencies of the clinical features between patients with PCNVs and previously reported patients with *H19*-hypo or UPD(7)mat [9, 29] (Table 2). The frequency of CHD was significantly higher in patients with PCNVs than in both patients with *H19*-hypo and patients with UPD(7)mat. Regarding the frequency of motor development, significant differences were not identified between two groups (PCNVs vs. *H19*-hypo and PCNVs vs. UPD(7)mat). The incidence of speech delay in patients with PCNVs was significantly higher than that in patients with *H19-*hypo, and there was no significance between patients with PCNVs and those with UPD(7)mat. In patients with UPD(7)mat, abnormal gene expression of *FOXP2* on chromosome 7 associated with language development may lead to speech delay [30]. All of the patients with PCNVs other than patient 2 presented moderate to severe global developmental delay, while most of the patients with *H19*-hypo and UPD(7)mat in a previous study presented with mild delay or normal range [16]. Our study suggests that patients with PCNVs, who have a phenotype resembling SRS, have a high tendency towards CHD or apparent developmental delay. To provide better treatment and genetic counseling, we suggest that attending physicians perform copy number analysis prior to methylation analysis for patients with the SRS phenotype together with CHD, apparent developmental delay, or other dysmorphic features.

Our study has some limitations. First, as several attending physicians including general pediatricians, neonatologists, pediatric endocrinologists, and pediatric geneticists, assessed patients’ clinical features, diagnostic bias may have occurred during clinical diagnosis for those items that were subjectively diagnosed, such as a protruding forehead, triangular face, fifth finger clinodactyly, and brachydactyly. In addition, since all attending physicians were not specialists in human genetics, clinical diagnoses for genetic syndromes may not be accurate. Second, although the NH-CSS is the most sensitive and has the highest negative predictive value among previously reported SRS clinical scoring systems, because most clinical features of SRS are not specific, the specificity of NH-CSS is low (36%), which is similar to that of other scoring systems [8]. As clinical diagnosis based on only NH-CSS might result in misdiagnosis with SRS, the evaluation of the clinical features other than NH-CSS items is also important for diagnosis. Ideally, the patients with the SRS phenotype should be evaluated by clinical geneticists prior to performing genetic testing.

In conclusion, 5.6% of SRS-compatible and 7.1% of SRS-like patients had PCNVs in our study. In addition, our study revealed the difficulties of diagnosing SRS in patients with severe neurological complications, and the need to consider mosaic trisomy 18, 19q13.11 deletion syndrome and atypical Williams syndrome for a differential diagnosis of SRS. Patients misdiagnosed with SRS who have PCNVs may have a high tendency towards CHDs and/or apparent developmental delay. To provide better treatment and genetic counseling for patients with the SRS phenotype together with CHD, apparent developmental delay, or other dysmorphic features, attending physicians should consider the alternative diagnoses or refer patients to clinical geneticists prior to performing genetic testing. Furthermore, if molecular analysis is required, copy number analysis should be considered before methylation analysis.

## Abbreviations

BMI: Body mass index
CHD: Congenital heart disease
DMR: Differentially methylated region
*H19*-hypo: Hypomethylation of the *H19*-DMR at the 11p15 imprinted region
LLD: Leg length discrepancy
NH-CSS: Nethine-Harbison clinical scoring system
PCNV: Pathogenic copy number variation
SDS: Standard deviation score
SRS: Silver-Russell syndrome
UPD(7)mat: Maternal uniparental disomy chromosome 7
